# Preparation and Performance Study of Composite Aramid Paper for High-Frequency Working Conditions

**DOI:** 10.3390/nano14231880

**Published:** 2024-11-22

**Authors:** Xiaonan Li, Tong Qin, Wenxu Zhang, Hong Wang, Yanhong Chen, Kangle Li, Qing Wang, Yibo Wang

**Affiliations:** 1 School of Electrical Engineering and Information Engineering, Lanzhou University of Technology, Lanzhou 730050, China; lixiaonan1989@163.com (X.L.); 222085801038@lut.edu.cn (T.Q.);; 2 School of Electrical Engineering, Northwest Minzu University, Lanzhou 730030, China

**Keywords:** high-frequency operating conditions, composite aramid paper, BNNS, compound particles

## Abstract

When the power converter connects to the high-frequency transformer breaks through the bottleneck and reaches a frequency of 100 kHz or even higher, the high-frequency transformer’s inter-turn insulation faces more serious high-frequency discharge and high-temperature problems. In order to improve the service performance of oil-immersed high-frequency transformer insulation paper, composite K-BNNS particles are prepared by ultrasonic stripping, heat treatment, and thermomagnetic stirring. Then, K-BNNS particles are mixed with PMIA (polymeric m-phenylenediamine solution) slurry to produce composite aramid paper. And the effects of K-BNNS particles with different contents on the thermal conductivity, dielectric properties, partial discharge properties, and mechanical properties of aramid paper are explored. It can be found that, when the addition of composite particles (K-BNNS) is 10%, the comprehensive performance of composite aramid paper is the best. Compared with Nomex paper, the in-plane and through-plane thermal conductivity of composite insulating paper F-10 increased by 668.33% and 760.66%, respectively. Moreover, the high-frequency breakdown voltage increased by 48.73% and the tensile strength increased by 2.49%. The main reason is that the composite particles form a complete thermal conductive network in the aramid paper matrix and a large number of hydrogen bonds with the matrix, which enhances the internal interface bonding force of the material and changes the charge transport mechanism.

## 1. Introduction

In recent years, the power system has been evolving towards a new system primarily based on wind and solar energy under the overarching goal of carbon neutrality in China. This shift has led to the gradual development of power transmission equipment towards a high proportion of power electronics. Consequently, core electrical equipment in power transmission equipment, such as high-frequency transformers, is now facing challenges related to high-frequency operation. These transformers, which are connected to power electronic converters, are subjected to repetitive square wave pulse voltages characterized by short rise times, high amplitudes, and high frequencies. Additionally, high-frequency transformers are also challenged by high power density and compact, lightweight designs, resulting in reduced volume and weight compared to their counterparts with similar capacities. These compact structures make heat dissipation more difficult in transformers. Notably, power converters connected to high-frequency transformers will break through technological bottlenecks in the future, achieving frequencies up to 100 kHz or even higher [1,2]. In addition, given their excellent cooling and insulating properties, oil-immersed transformers enable the development of high-frequency transformers towards high power, high voltage, and large capacity. Aramid paper, used as the primary inter-turn insulation material in oil-immersed high-frequency transformers, is prone to partial discharge and local overheating, which are principal causes of premature failure of inter-turn insulation [3,4,5]. Therefore, there is an urgent need to develop composite aramid paper with excellent resistance to high-frequency partial discharge and high temperatures.

Currently, the methods for enhancing the performance of insulating materials primarily include the preparation of intrinsic materials and filled composite materials. In the field of intrinsic insulating materials, Huang et al. [6] proposed that incorporating phenyl sulfide groups into polyimide molecular chains can effectively reduce the high-frequency dielectric loss of the modified polyimide films. Wang et al. [7] investigated the effects of vapor phase fluorination on the surface charge and space charge characteristics of oil-immersed Nomex paper. They found that vapor phase fluorination accelerates the decay rates of both positive and negative charges and reduces the energy and density of hole and electron traps in the fluorinated samples. However, the preparation of intrinsic materials is costly and challenging to industrialize.

In the realm of filled composite materials, the primary approach involves adding high thermal conductivity and high insulation fillers to the insulating material matrix to enhance the material’s overall performance, including discharge resistance, high-temperature resistance, and corona resistance. Common fillers used include one-dimensional, two-dimensional, and three-dimensional fillers. Duan et al. [8] utilized reduced graphene oxide (frGO) and one-dimensional multi-walled carbon nanotubes (MWCNTs) as mixed thermal conductive particles to increase the density of thermal conductive network within the aramid insulation paper matrix. The thermal conductivity of the aramid paper (PMIA) is effectively improved, and the insulation properties of the composite insulating materials are declined due to the high conductivity of carbon materials. Liu et al. [9] incorporated functionalized two-dimensional boron nitride nanosheets (BNNS) into the polyetherimide (PEI) matrix. They found that adding an appropriate amount of BNNS enhances the breakdown strength of the matrix due to the formation of dispersed deep traps within the material. Qin et al. [10] prepared BN fiber–graphene microplatelets using the melt blending method. They found that the thermal conductivity is 4.2 times higher than that of polypropylene (PP), and electrical insulation properties are slightly improved, attributed to the unique “dual network” structure formed by GNP and BN fibers. Liu et al. [11] added two-dimensional BN to silicone rubber, achieving a 30% increase in thermal conductivity as well as improvements in electrical breakdown strength and hydrophobicity, though the mechanical performance deteriorated. Huang et al. [12] prepared three-dimensional boron nitride nanosheet/epoxy composites via radial freeze casting, resulting in composite materials with through-plane thermal conductivity of 4.02 W/(m·K) and in-plane thermal conductivity of 3.87 W/(m·K). Chen [13] employed electrospinning to fabricate three-dimensional thermal conductive networks within polyvinylidene fluoride (PVDF) polymer nanocomposite films by filling them with BNNS, achieving ultra-high in-plane thermal conductivity and excellent electrical insulation properties. However, the production costs of three-dimensional fillers are high, and the preparation process is complex [14]. Additionally, it is crucial to note that agglomeration occurs when the nano-filler content is high or the compatibility between the filler and the matrix is poor, adversely affecting the various properties of the insulating materials [15].

Existing research shows that the addition of coupling agents can effectively improve the compatibility between nano-fillers and the matrix. Zha et al. [16] used KH-550 silane coupling agent to modify and prepare PI/TiO_2_ nanocomposite films. The coupling agent was found to effectively improve the compatibility between the nano-filler and the matrix, resulting in a more uniform dispersion in the matrix. Lv et al. [17] studied the effect of different types of silane coupling agents on the electrical properties of meta-aramid composite insulating paper with nano-TiO_2_. It can be found that suitable types of silane coupling agents can effectively improve the dispersion of nano-fillers in the matrix, increasing the breakdown voltage and volume resistivity of the composite insulating paper. Additionally, these agents enhance the temperature resistance and mechanical strength of aramid fibers.

The aforementioned studies primarily focus on the thermal conductivity of modified insulating materials. However, some researchers have also investigated the changes in the high-frequency discharge resistance of modified composite insulating materials. For example, Li et al. [18] found that adding dopamine-grafted nano boron nitride to epoxy resin can effectively improve the charge dissipation rate and flashover voltage of epoxy resin composite material under high-frequency conditions. Zhao et al. [19] weakened the electron trap capture effect under high-frequency conditions by pre-impregnating GHG paper, effectively reducing the flashover voltage. Li et al. [20] studied the high-frequency partial discharge characteristics at different aging stages of epoxy resin. It can be found that the amount and number of discharges generally increase as the probability of effective initial electron generation on the surface of insulation defects changes, and the phase range of partial discharge extends towards both ends. Currently, research on the performance of composite insulating materials under high-frequency conditions is limited and requires further exploration. In view of the above results, it is found that there is still a lack of comprehensive studies on the partial discharge resistance and high-temperature characteristics of composite insulating papers under high-frequency conditions. Therefore, such investigations are carried out here on an aramid paper nanocomposite made by exfoliating boron nitride nanosheets (BNNS) using heat treatment and ultrasonic exfoliation and then modifying them with a silane coupling agent KH-550. The results show that composite aramid paper has the best thermal conductivity and partial discharge properties at high frequency when the filler content is 10%.

## 2. Experimental Section

### 2.1. Main Materials

The materials used in this study are shown in Table 1.

### 2.2. Preparation of High-Performance Composite Aramid Paper

#### 2.2.1. Preparation Process of Boron Nitride Nanosheets (BNNS)

##### Preparation of BNNS

A 600 mg quantity of h-BN (hexagonal boron nitride) is added to a round-bottom flask containing 100 mL of double-distilled water. The mixture is sonicated for 2 h and then allowed to precipitate for 1 h. After complete precipitation of h-BN, the h-BN was separated. The h-BN is then heat-treated at 400 °C for 2 h. After cooling to room temperature, the sample is dispersed in water for 2 h and then allowed to precipitate for 12 h. The upper clear liquid precipitated is BNNS [21]. The upper clear liquid is filtered using a polytetrafluoroethylene membrane (pore size: 0.1 μm). The remaining product filtered is dried in a vacuum oven at 90 °C for 20 h, obtaining BNNS.

##### Preparation of BNNS-OH

The BNNS is heated to 1000 °C and maintained for 1 h. After cooling, the material is washed with hot water and dried, obtaining hydroxylated boron nitride nanosheets (BNNS-OH) [22].

#### 2.2.2. Preparation Process of Composite Filler KH-550@BNNS Particles

A 4% mass fraction of KH-550 is prepared, with the solvent being a mixture of deionized water and anhydrous ethanol in a 9/1 ratio. The mixture is reacted at 80 °C for 30 min. Then, 200 mL of a 2% BNNS-OH solution is added. The mixed solution is magnetically stirred at 80 °C for 5 h and is washed multiple times with deionized water and anhydrous ethanol, and filtered to remove unreacted silane coupling agents. Finally, it is baked in a vacuum at 90 °C for 10 h, obtaining KH-550-modified BNNS, denoted as K-BNNS.

#### 2.2.3. Preparation Process of Composite Aramid Paper

**Figure 1:**
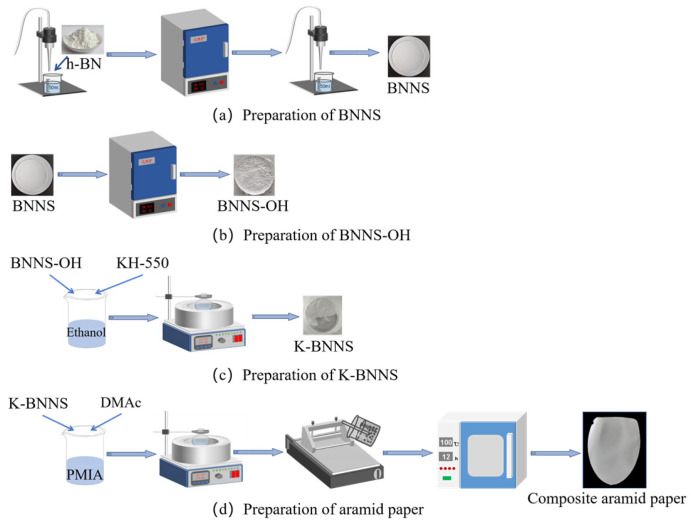
Flow chart of the preparation of composite aramid paper.

The PMIA slurry was diluted to a 12% solid content (using DMAc as the solvent). KH-550@BNNS are dispersed in DMAc and added to the PMIA slurry. The mixture is stirred at 50 °C for 6 h and then degassed under vacuum for 1.5 h. The KH-550@BNNS/PMIA wet film is prepared using a coating machine. The wet film is first placed in an oven at 100 °C for 16 h, then transferred to a vacuum oven at 80 °C, and dried for 16 h to completely remove the DMAc solvent. Composite aramid papers with particle contents of 0%, 5%, 8%, 10%, and 13% are denoted as F-0, F-5, F-8, F-10, and F-13, respectively. The preparation process is shown in Figure 1. Different performance tests are conducted on composite aramid paper samples, with each test repeated five times.

## 3. Results and Discussion

### 3.1. Performance Characterization of Composite Particles

#### 3.1.1. FTIR Analysis

**Figure 2:**
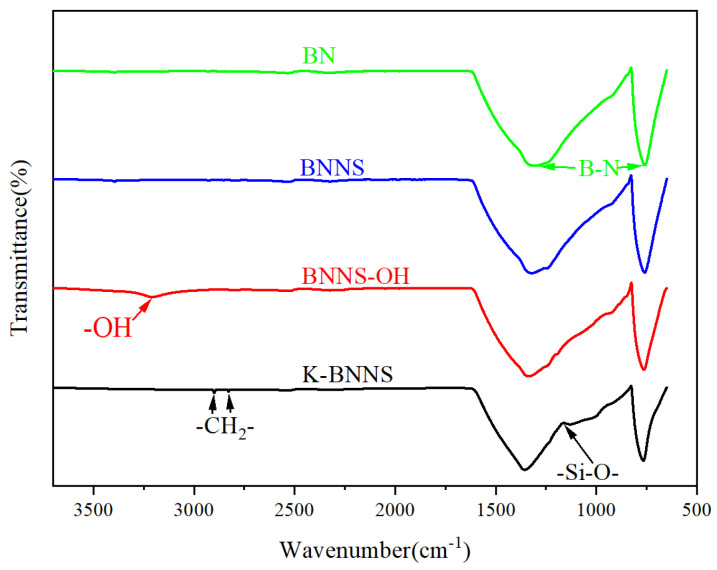
FTIR spectra of different samples.

FTIR is used for the chemical structure analysis of the particles. The results are shown in Figure 2. It can be observed that the characteristic absorption peaks of B-N bonds appeared in all four types of particles. For BNNS-OH particles, a distinct hydroxyl (-OH) absorption peak is observed at 3208.95 cm^−1^. This indicates that hydroxyl groups are successfully grafted onto the surface of BNNS after high-temperature treatment.

In the K-BNNS particles, the hydroxyl characteristic absorption peak completely disappeared due to the reaction between KH-550 and the hydroxyl groups of BNNS-OH particles. In addition, new characteristic absorption peaks of methylene (-CH_2_-) appeared at 2830.38 cm^−1^ and 2900.58 cm^−1^, and a characteristic absorption peak of Si-O bonds was observed at 1131.77 cm^−1^. These findings confirm that the silane coupling agent KH-550 undergoes coupling reactions with BNNS-OH, successfully grafting silane onto the surface of BNNS-OH.

#### 3.1.2. XRD Analysis

**Figure 3:**
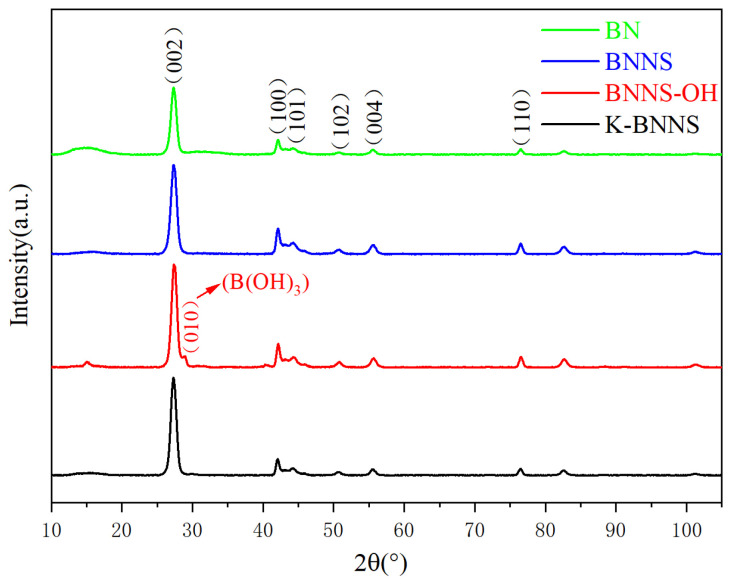
XRD spectra of BN, BNNS, BNNS-OH, and K-BNNS.

The crystal structures of BN, BNNS, BNNS-OH, and K-BNNS are analyzed by XRD. The results are shown in Figure 3.

As seen from this figure, the XRD patterns of the four types of particles are generally similar. The characteristic peaks of BN crystals at 26.749°, 41.583°, 43.692°, 50.077°, 54.935°, and 76.082° correspond to the diffraction peaks of the (002), (100), (101), (102), (004), and (110) crystal planes, respectively. The characteristic peak intensity of BNNS obtained by heat treatment combined with ultrasonic exfoliation is higher than that of BN, indicating that this exfoliation method produces well-developed BNNS crystals.

Additionally, the XRD pattern of BNNS-OH shows a diffraction peak of B(OH)_3_ at the (010) crystal plane at 28.94°, suggesting successful grafting of hydroxyl groups onto the surface of BNNS after high-temperature treatment. This finding is consistent with the FTIR analysis results.

#### 3.1.3. TEM Analysis

**Figure 4:**
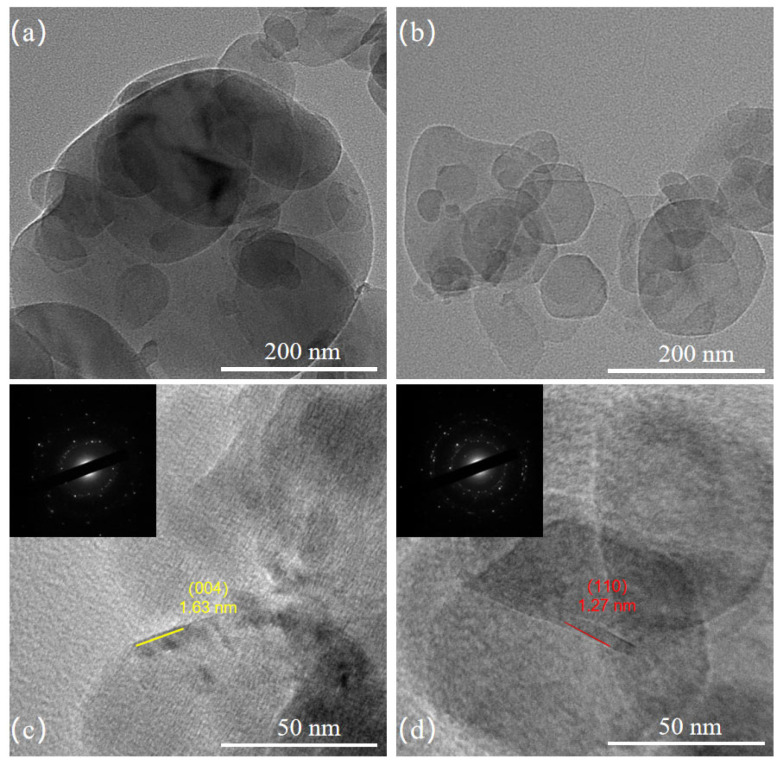
(**a**,**b**) are TEM patterns of BN and BNNS; (**c**,**d**) are electron diffraction patterns of BN and BNNS.

TEM is used to analyze BN and BNNS particles. The results are shown in Figure 4. It can be seen that, for BN particles, multiple layers are clearly stacked due to intermolecular forces. For BNNS, the multilayer structure disappears as a result of heat treatment and ultrasonic exfoliation, making BNNS appear semi-transparent. Additionally, the size of BNNS ranging from 100 nm to 50 nm is slightly smaller compared to BN, and the large-sized plate-like structures disappear. Some small nanosheets might originate from defects in the precursor BN or be inherited from small BN fragments.

Figure 4c,d show that the lattice spacing of the (004) crystal plane in BN crystals is 1.63 nm, while the lattice spacing of the (110) crystal plane in BNNS crystals is 1.27 nm. Comparing these measurements with the standard BN PDF card reveals a slight increase in lattice spacing after exfoliation. Comparing the electron diffraction patterns in the top left corners of Figure 4c,d, it can be concluded that exfoliation causes minor structural changes. The electron diffraction pattern in Figure 4c clearly shows a six-fold symmetric structure, while the pattern in Figure 4d only shows a symmetric arrangement due to layer overlap.

### 3.2. Performance Characterization of Composite Aramid Paper

#### 3.2.1. SEM and EDS Analysis

**Figure 5:**
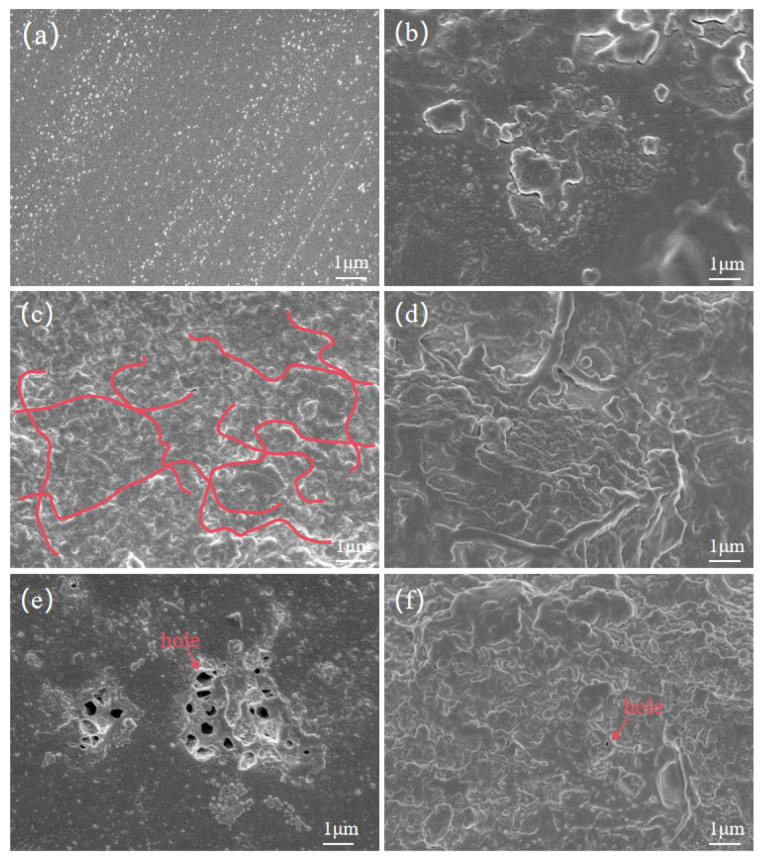
(**a**) F-0 surface; (**b**) F-0 cross-section; (**c**) F-10 surface; (**d**) F-10 cross-section; (**e**) F-10 surface not modified with KH-550; and (**f**) F-10 cross-section not modified with KH-550.

Based on Section 1, a cold-field emission scanning electron microscope is used to analyze composite aramid paper with different contents of highly insulating and thermally conductive particles: 0%, 10%, and 10% without KH-550 modification. The surface and cross-sectional microstructure characteristics are obtained and shown in Figure 5.

From this figure, it is evident that the surface of F-0 is smooth, with small, agglomerated protrusions on the cross-section. The surface of F-10 becomes rough, and the cross-section shows interlaced dendritic and layered structures. This indicates that the addition of K-BNNS improves the agglomeration phenomenon during the coating and forming process of aramid pulp, leading to an interlaced, complex dendritic and layered structure. These structures make the internal matrix of aramid paper more uniform and the connections between particles tighter, facilitating the formation of a thermal conductive network within the composite aramid paper, hence increasing thermal transfer efficiency.

To demonstrate the effect of KH-550 on improving the compatibility of modified particles with the aramid paper matrix, the aramid paper with 10% unmodified particle content is observed as shown in Figure 5e,f. It is evident that this sample not only has voids on the surface but also shows a few voids at the cross-section. Additionally, severe surface agglomeration is observed, and a clumped morphology is displayed in the cross-section, which may be less effective in establishing a thermal conductive network compared to the dendritic structure of F-10.

Therefore, it can be concluded that the addition of K-BNNS particles significantly improves the microstructure of aramid paper. Not only does it result in a more uniform organization, but also the interlaced dendritic and layered microstructure is highly beneficial for establishing a thermal conductive network within the aramid paper. Moreover, the KH-550-modified BNNSs-OH greatly improves the compatibility between the thermally conductive particles and the matrix, significantly reducing agglomeration within the material and resulting in a denser and more uniform structure, leading to effective modification.

**Figure 6:**
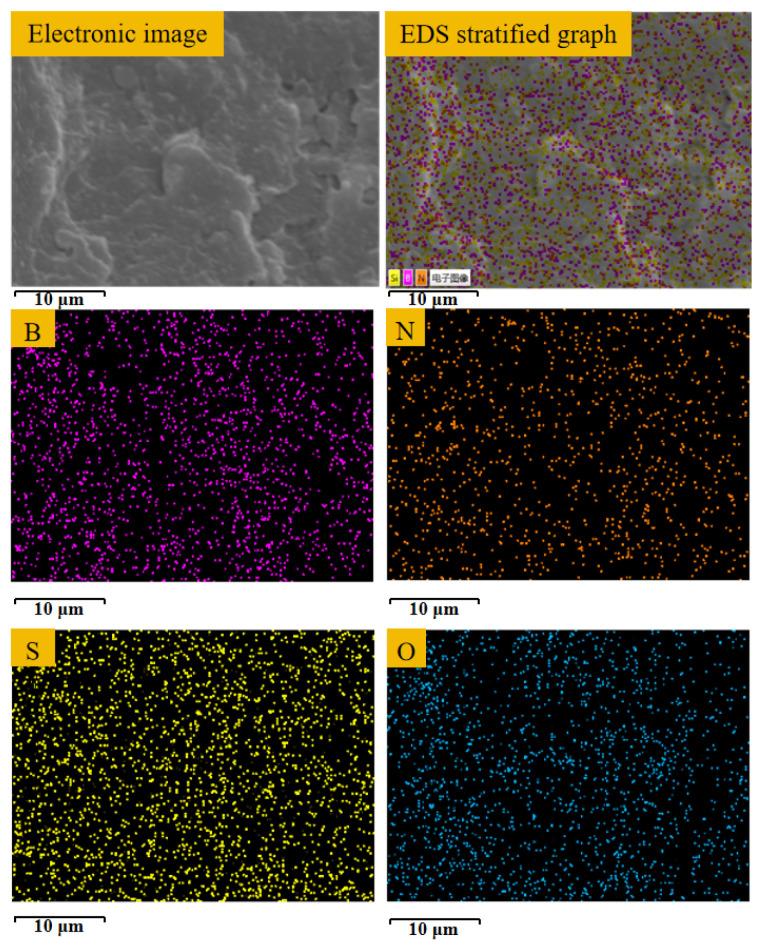
EDS analysis of F-10 composite aramid paper selection.

To further confirm the incorporation of K-BNNS particles into the composite aramid paper, selected area EDS analysis is performed on the cross-section of F-10, and the obtained results are shown in Figure 6. It can be observed that the elements of B, N, Si, and O are uniformly distributed across the cross-section. This proves that the composite aramid paper contains not only boron nitride nanosheets but also KH-550. Thus, the successful addition of K-BNNS particles is demonstrated.

#### 3.2.2. Thermogravimetric and Thermal Conductivity Analysis

**Figure 7:**
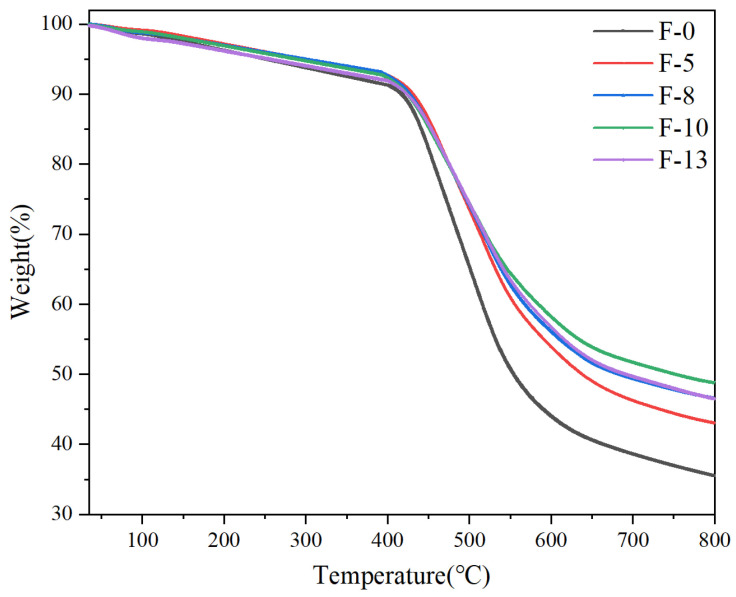
TGA curves of composite aramid paper with different contents of K-BNNS.

Thermogravimetric analysis (TGA) is performed on composite aramid paper with different K-BNNS contents, and the obtained results are shown in Figure 7. The TGA curves indicate that the composite aramid papers containing modified particles exhibit similar trends and better thermal stability compared to pure meta-aramid paper. Within the range of 380–430 °C, small molecules within the composite aramid paper decompose. Between 430 and 540 °C, a significant mass loss occurs in meta-aramid paper, likely due to increased molecular thermal motion as temperature rises, where impurity molecules in the air penetrate the aramid paper and react. The reaction causes cleavage of C-N and C-O bonds and the subsequent gradual thermal degradation of unstable macromolecular chains.

As the temperature further increases, molecular motion within the aramid paper becomes more pronounced, and the previously undecomposed macromolecular chains gradually degrade into small molecular chains. By 800 °C, the residual rates of F-0, F-5, F-8, F-10, and F-13 are 35.57%, 43.07%, 46.56%, 48.86%, and 46.53%, respectively, with F-10 achieving the highest residual rate. This indicates that the addition of the highly thermally conductive and insulating K-BNNS reduces the degradation rate of aramid paper throughout the thermal decomposition process, particularly mitigating fiber decomposition significantly in the range of 400–800 °C. The enhanced thermal stability is primarily attributed to the exceptional thermal stability of the added composite particles, making the material less prone to decomposition at high temperatures.

**Figure 8:**
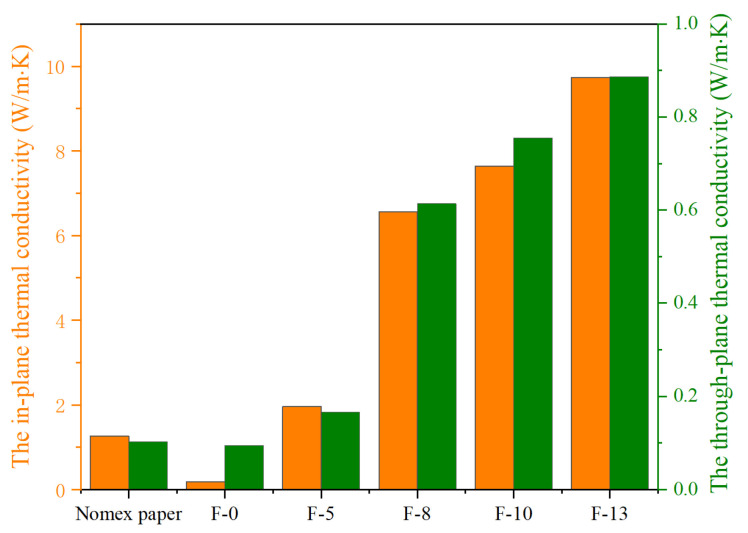
In-plane and through-plane thermal conductivity of different samples.

Thermal conductivity measurements are performed on composite aramid papers with varying K-BNNS contents and Nomex paper, analyzing both in-plane and through-plane thermal conductivity. The results are shown in Figure 8.

It can be observed that both the in-plane and through-plane thermal conductivity of the composite aramid papers containing K-BNNS particles are superior to that of pure meta-aramid paper. Additionally, the thermal conductivity increases with the addition of nano-fillers. When the nano-filler content reaches 13%, the composite aramid paper exhibits the highest in-plane and through-plane thermal conductivity values, which are 9.73 W/(m·K) and 0.89 W/(m·K), respectively. Compared to Nomex paper, the in-plane thermal conductivity improves by 668.33%, and the through-plane thermal conductivity improves by 760.66%. Compared to other composite insulating papers prepared by existing researchers [23,24,25,26,27], as shown in Table 2, the composite insulating paper prepared in this study exhibits better thermal performance at lower filler contents.

This enhancement is attributed to the good compatibility of boron nitride nanosheets with the matrix material within the composite aramid paper, leading to the construction of thermal conductive networks within the material. When the nano-filler content is low, the conductive particles may be relatively dispersed within the material, with small, independent thermal conductive chains that do not link to form continuous pathways, thus offering limited improvement in thermal conductivity. However, with further increases in thermal conductive filler content, more thermal conductive molecular chains appear within the material. These chains begin to contact and link with each other, forming a dense, intertwined thermal conductive network that significantly enhances the thermal conductivity of the composite aramid paper.

#### 3.2.3. Dielectric Properties Analysis

**Figure 9:**
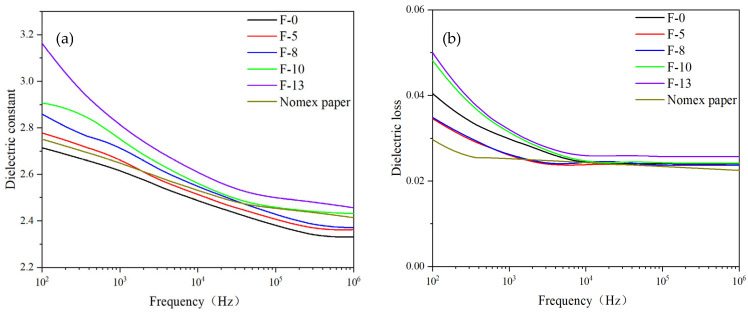
Dielectric constant and dielectric loss for different samples: (**a**) dielectric constant and (**b**) dielectric loss.

Dielectric parameters are tested using a dielectric spectrometer for different samples. The results are shown in Figure 9.

From Figure 9a, it can be seen that the dielectric constant of all samples increases with the content of K-BNNS and slightly decreases with an increase in the test frequency. This may be because the intrinsic dielectric constant of BN is approximately 4, which is higher than that of PMIA. Hence, a higher K-BNNS content significantly enhances the dielectric constant of the composite material. Additionally, the modification of BNNS-OH particles with KH-550 increases the number of polar groups in the composite aramid paper matrix, leading to a higher dipole density and enhanced polarization capability, thus increasing the dielectric constant.

From Figure 9b, it is observed that the dielectric loss of all samples decreases as the frequency increases, and this is because the dipole polarization loss is obvious at a low frequency, and the dipole is hard to turn at a high frequency. The dipole polarization loss decreases, and the dielectric loss is determined by the conductance loss. The dielectric loss of the composite aramid paper decreases initially and then increases with the rise in K-BNNS content. This trend is possibly due to the fact that the K-BNNS particles at higher K-BNNS content begin to contact each other within the PMIA matrix. At high concentrations (F-10 and F-13), the nanosheets become close enough to provide linked paths for carrier transport. This contact leads to conductive loss, which contributes to the dielectric loss in proportion to the frequency, thereby increasing the dielectric loss.

#### 3.2.4. Partial Discharge (PD) Characteristics Analysis

**Figure 10:**
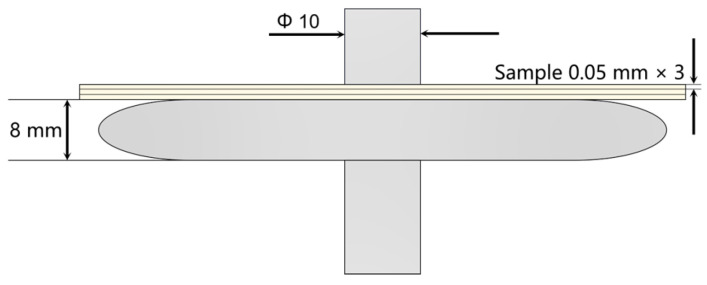
Typical column-plate model.

**Figure 11:**
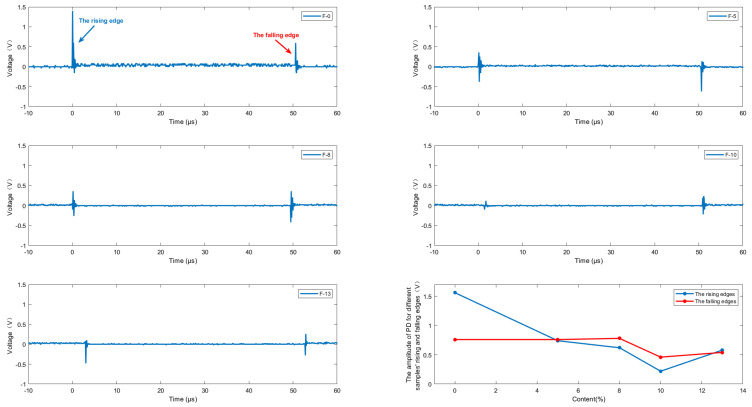
The amplitude of PD.

**Figure 12:**
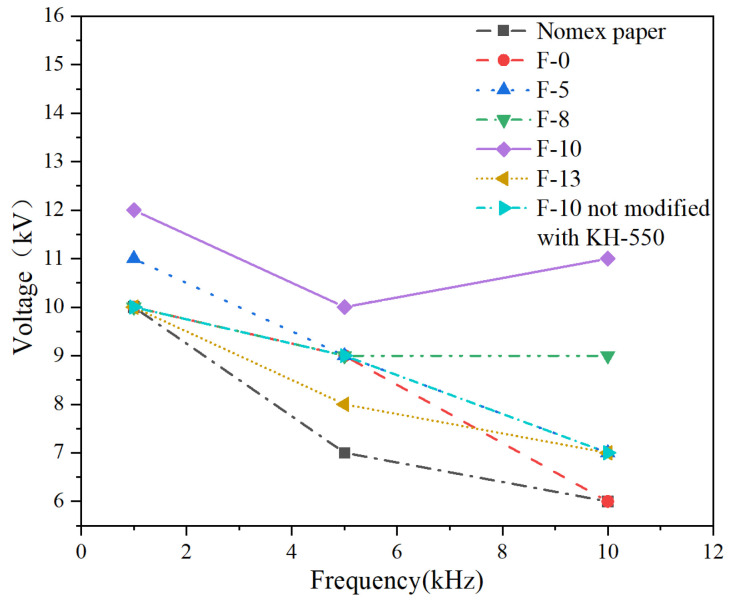
Breakdown voltage of PD in different samples under high-frequency voltages.

According to the standard GB/T1408.1-2006 [28], a typical defect model of transformer is designed as shown in Figure 10. In this model, a step-up voltage method is applied to conduct high-frequency partial discharge tests. The results are shown in Figure 11 and Figure 12.

**Table 3:** Insulation material parameter index [29].

Insulating Material	Model Number	Nominal Thickness/mm	Heat Resistance Level	Calculate the Intensity of Electric Field/(kV/mm)
DMD paper	6641F	0.30	F	≥30
PET membrane film	6020	0.125	E	≥100
PI membrane film	6050	0.10	H	60~100

**Figure 13:**
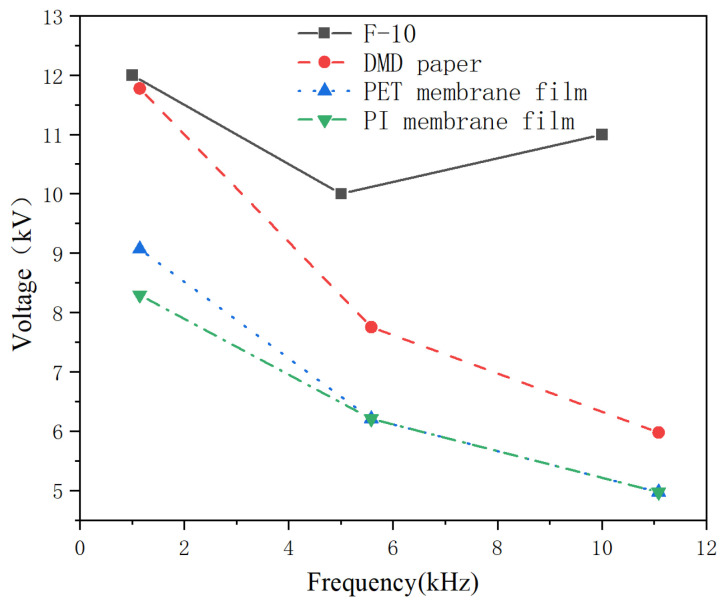
Breakdown voltage of PD in different materials [30].

Figure 11 shows the partial discharge amplitude of composite aramid paper with different filler contents at 5 kHz and 5 kV. The discharge amplitude decreases initially and then increases with increasing filler content, and the discharge amplitude of F-10 sample is the smallest. This indicates that the composite aramid paper with 10% filler content has the best discharge resistance. As seen in Figure 12, the breakdown voltage of F-10 sample at different frequencies is higher than that of other composite aramid papers. Compared to Nomex paper, the breakdown voltage increases by an average of 48.73%. Compared with other composite materials prepared by existing researchers, as shown in Table 3 and Figure 13, the F-10 sample prepared in this study exhibits a significant improvement in partial discharge breakdown voltage.

**Figure 14:**
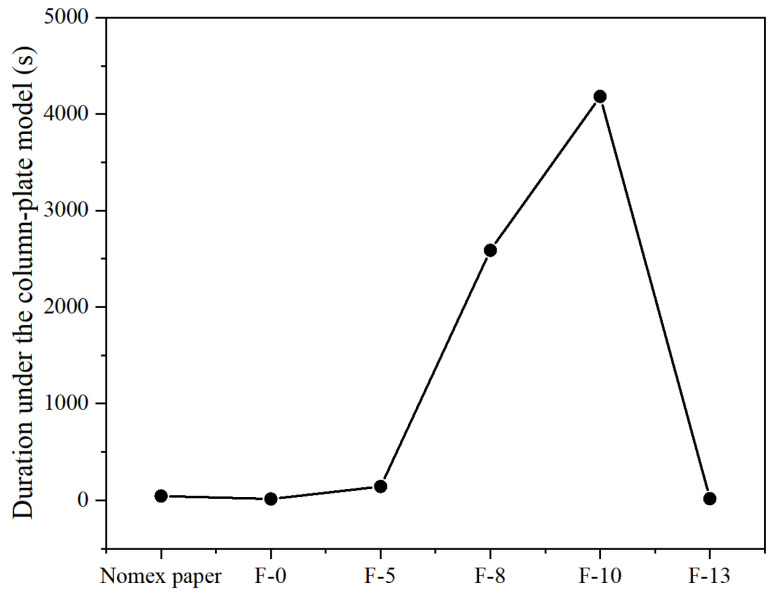
The duration of partial discharge voltage resistance of different materials at 20 kHz.

The duration of discharge resistance of the composite insulation paper is tested using a constant voltage method under high frequency voltage at 20 kHz and 5 kV. The results are shown in Figure 14. It is observed that the duration of discharge resistance for F-10 sample is the longest, which correlates with the results of the partial discharge breakdown voltage test.

In summary, the F-10 sample exhibits the best partial discharge resistance. This is attributed to the formation of a thermal conductive network and the improvement in interfacial bonding ability between the matrix and fillers. On the one hand, a thermal conductive network is constructed within the composite aramid paper due to high-thermal conductivity insulating particles (K-BNNS). As a result, the interfacial thermal resistance of the matrix is increased, phonon scattering loss during transmission is reduced, the heating rate of the insulation material is declined, and the trapping and de-trapping process of active electrons is slowed down. It means that the degree of electric field distortion at the insulation defect is reduced, further inhibiting the generation of partial discharge (shown in Figure 11–Figure 14). On the other hand, the interfacial bonding ability between the matrix and fillers increases, the internal defects and traps of the material are reduced. It makes the electrons difficult to de-trap and suppresses the mobility of charge carriers even at larger electric field strengths, thereby suppressing the occurrence of partial discharge.

#### 3.2.5. Analysis of Space Charge Characteristics

**Figure 15:**
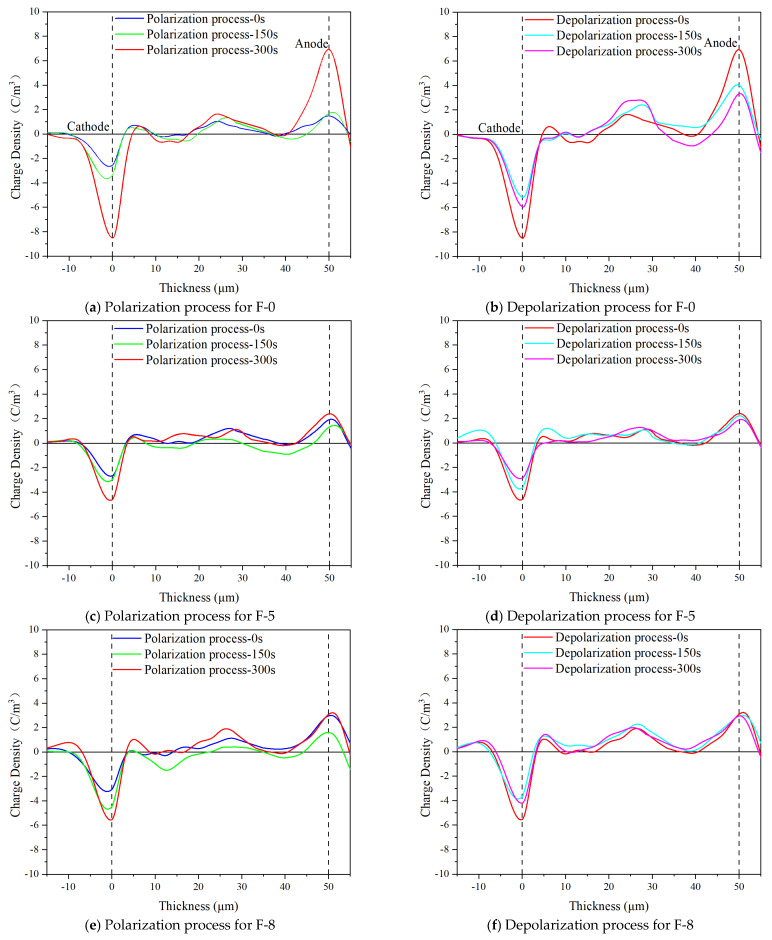

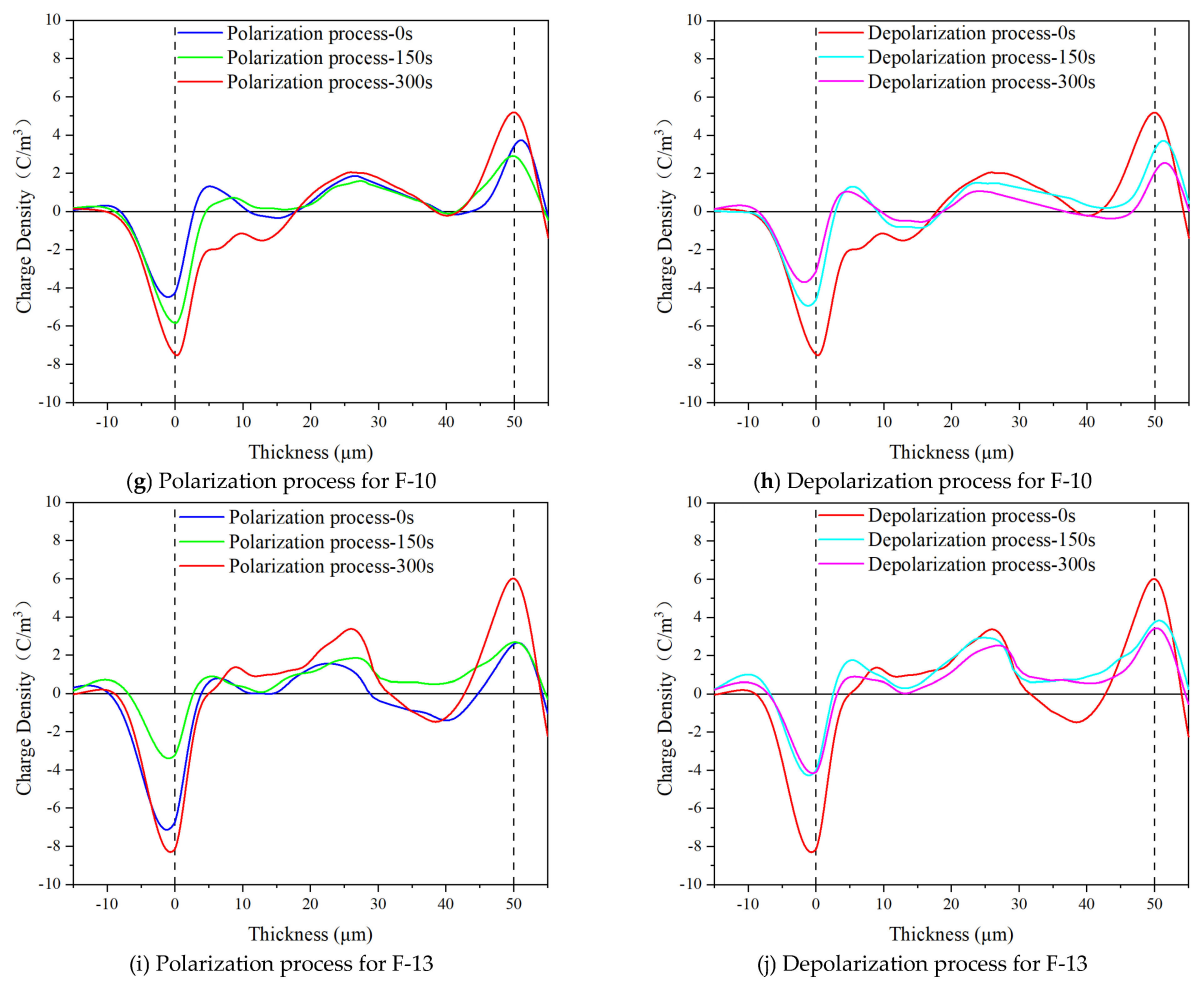
Space charge curves of different insulation papers under 10 kV/mm field strength.

The pulsed electro-acoustic method is used to investigate the effect of K-BNNS on charge migration during the polarization process of composite aramid paper, and different samples are subjected to an electric field strength of 10 kV/mm. The result of space charge curves obtained are shown in Figure 15. It is observed that all samples exhibit charge injection during the polarization process, while charges migrate to the material surface and interior during the depolarization process, accompanied by recombination and dissipation processes. By reminding us of the variation in the amplitude of the spatial charge peak shown in Figure 15, the true spatial charge distribution map requires us to transform the data.

During the polarization phase, the amount of charge accumulation in the composite aramid paper significantly decreases when the K-BNNS content is relatively low (0–8%). The charge accumulation in the F-13 sample is similar to that in the F-0 sample compared to the other samples. During the depolarization phase, the charge dissipation also decreases. However, with an increasing content of composite particles (10–13%), the charge accumulation during the polarization phase increases, as does the charge dissipation during the depolarization phase. This could be because K-BNNS possess high thermal conductivity insulation properties, enhancing the heat dissipation capability of the composite aramid paper and reducing the internal heat accumulation. The above reason makes charge trapping, de-trapping, and migration more difficult, which limits the charge transport process within the composite aramid paper. However, the addition of K-BNNS enhances the number of polar groups within the composite aramid paper, thereby increasing the rate of change in space charge density during the polarization process.

The change in charge density with thickness of composite aramid paper reflects the problem pertaining to the mechanism of the influence of different filler contents on the charge migration process. When the filler content is low (F-0–F-8), the migration ability of the spatial charge is significantly weakened. And when the filler content is high (F-8–F-13), the spatial charge gradually becomes active inside the material, but still lesser than F-0. This is mainly because of the addition of the filler in the composite aramid paper, which improves the internal defects of the material and suppresses the charge migration. But the dipole is introduced, which shows that the higher the filler content is, the more active the charge migration is. Therefore, in the process of obtaining a better thermal conductivity and insulation performance of composite aramid paper, the filler contents should not be too large.

#### 3.2.6. Mechanical Performance Analysis

**Figure 16:**
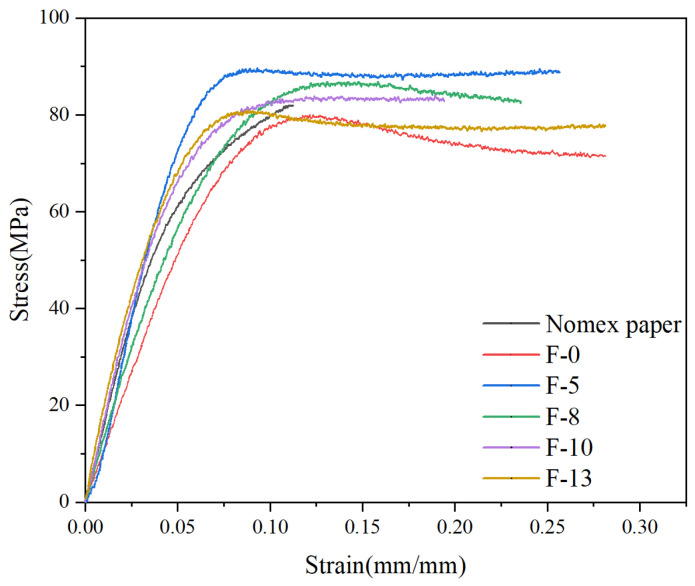
Stress–strain curves for different samples.

A universal testing machine is used to conduct tensile tests on different samples. The results are shown in Figure 16.

It can be seen that the composite aramid paper with 5% K-BNNS nano-filler exhibits the best mechanical performance, with a tensile strength of 89.69 MPa. As the nano-filler content increases, the mechanical performance gradually declines, but all samples still outperform pure meta-aramid paper. This may be attributed to the addition of minor amounts of well-compatible, small-volume second-phase particles, which can effectively improve internal defects in the matrix, such as pores and bubbles, thereby increasing the material’s density. However, with the further increase in nano-filler content, the volume fraction of filler particles in the material rises, leading to a rougher contact surface between the nano-filler and the matrix and a decline in mechanical performance.

## 4. Mechanism Analysis

### 4.1. Influence of Thermal Conductive Network on the Performance of Composite Aramid Paper

**Figure 17:**
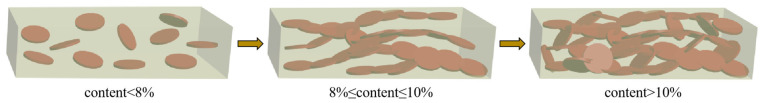
Thermal conduction network evolution.

As the content of the composite particles, K-BNNS, increases, a complete thermal conductive network gradually forms within the composite aramid paper, as shown in Figure 17. When the composite particle content is low (<8%), the particles cannot form thermal pathways within the matrix and are merely dispersed throughout the material. This results in a minimal improvement in the thermal conductivity of the insulating paper, which is consistent with the analysis results shown in Figure 8.

When the composite particle content is between 8% and 10%, the particles form a complete thermal conductive network within the matrix. One reason is that the composite particles show a high intrinsic thermal conductivity and constitute a complete thermal conductivity network, which is conducive to the heat propagation inside the material. This provides a path for the propagation of molecular thermal vibration phonons and improves the average free travel of phonons inside the material. It presents that the thermal conductivity increases significantly, the heat dissipation capacity of the material improves, the heat concentration inside the material reduces, and the deterioration of various materials is inhibited. The other reason is that the increased number of thermal conductive networks within the composite aramid paper decreases the interfacial thermal resistance of the matrix. This prevents excessive heat accumulation within the insulating material, partially suppressing the movement of active electrons under an external electric field. It means that the field intensity concentration is reduced, and the partial discharge activity is inhibited. However, as the composite particle content exceeds 10%, particle aggregation may occur within the aramid matrix, leading to an increase in defects and promoting partial discharge, as consistent with the results shown in Figure 12.

### 4.2. Influence of Hydrogen Bonds on the Performance of Composite Aramid Paper

**Figure 18:**
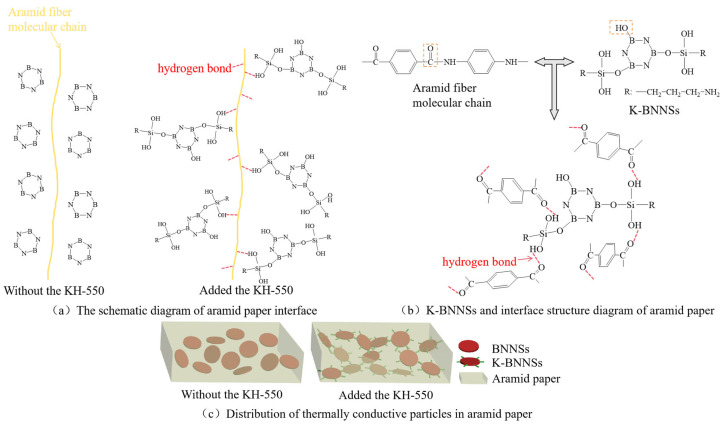
Schematic diagram of the interface of composite aramid paper.

**Figure 19:**
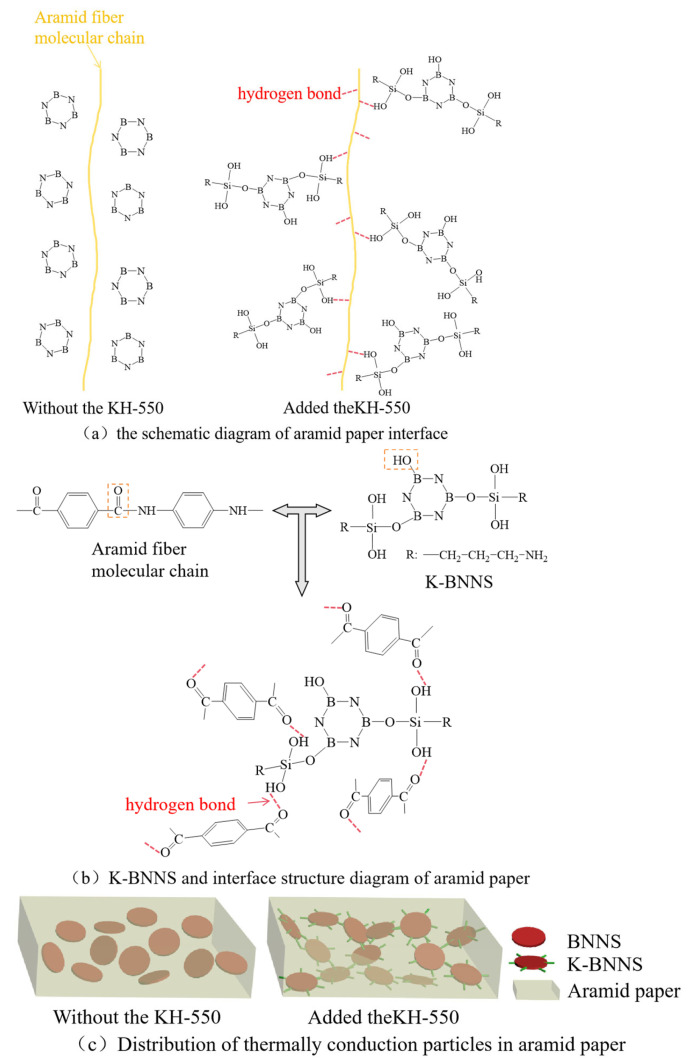

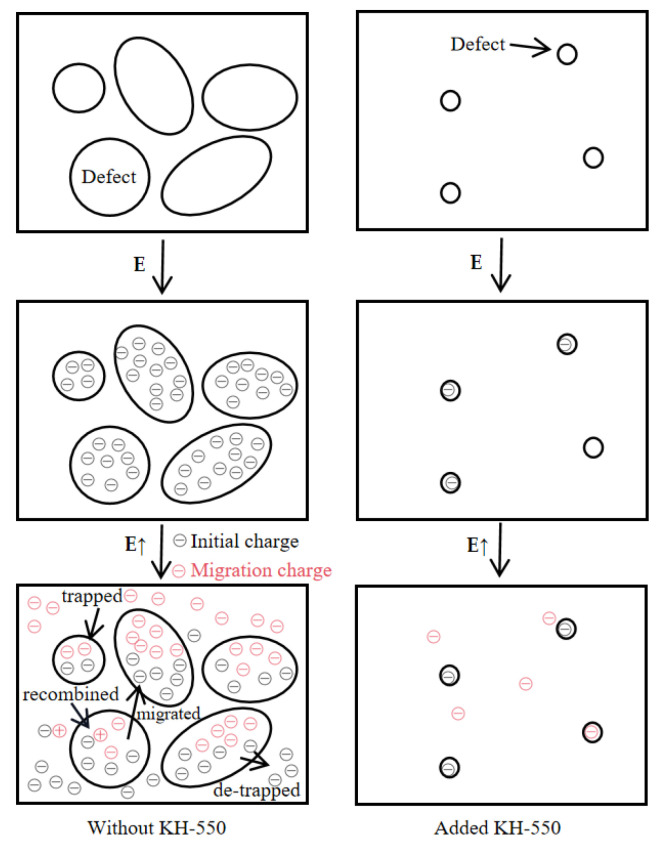
The effect mechanism of KH-550 on partial discharge.

Due to the absence of KH-550-modified particles, the compatibility between the composite aramid paper matrix and the composite filler particles is poor. This is evident from the lack of tight connections between the filler molecules and aramid fiber molecules, as well as among the filler particles themselves (see Figure 18). Consequently, numerous defects are present in the composite aramid paper, as shown in Figure 5e. When an electric field is applied, charge carriers are easily trapped in these defects, as indicated in Figure 19. Upon reaching the critical field strength, partial discharge readily occurs at these defect sites. Further increasing the field strength enhances the probability of trapping, de-trapping, migration, and recombination of charge carriers at these defects, accelerating the generation of partial discharge as seen in Figure 11 to Figure 14.

By adding KH-550, the BNNS-OH particles are functionalized, so that a large number of hydroxyl groups are filled around the composite particles. These hydroxyl groups readily form hydrogen bonds with aramid fiber molecules (see Figure 18), enhancing the interfacial bonding force between the matrix and composite particles, and thereby reducing defects in the composite aramid paper (see Figure 5c). In addition, defects are decreased in composite aramid paper and carrier migration is suppressed, which reduces partial discharges (see Figure 19). When an electric field is applied, the molecular chains within the material are less likely to be disrupted, inhibiting the occurrence of partial discharge. This improvement results in higher breakdown voltage and longer duration of discharge resistance.

### 4.3. Influence of Charge Transport on the Performance of Composite Aramid Paper

**Figure 20:**
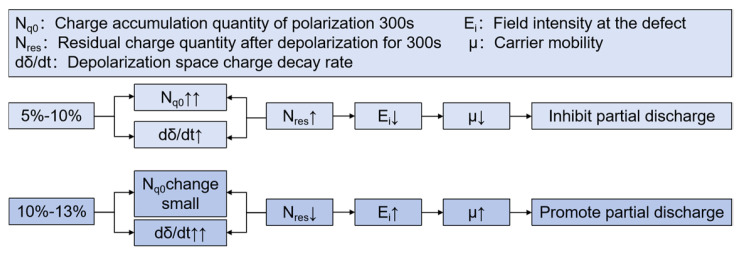
Charge transport mechanism.

The mechanism of space charge transport in composite aramid paper is analyzed in Figure 20. When the filler particle content is between 5% and 10%, both the charge accumulation during polarization and the charge decay rate during depolarization increase with higher filler content. The increase in charge accumulation is more pronounced, leading to a higher residual charge amount. It indicates that the electric field intensity at defect is reduced, carrier mobility is decreased, and partial discharge is suppressed.

When the filler particle content is between 10% and 13%, the charge accumulation during the polarization stage does not change significantly with increased filler content. However, the charge decay rate during the depolarization stage further increases, reducing the residual charge amount. It indicates that the electric field intensity at defect is increased, carrier mobility is increased, and partial discharge is promoted. As a result, the amplitude of partial discharge is increased, and the duration and breakdown voltage of partial discharge are lowered, which is consistent with the partial discharge characteristics analysis in Figure 11 to Figure 14.

## 5. Conclusions

(1) Through characterization methods such as FTIR, XRD, and TEM analysis, it has been confirmed that boron nitride nanosheets (BNNS) have been successfully exfoliated and modified by a silane coupling agent. A comparison between composite aramid papers containing BNNS and K-BNNS particles reveals that the silane coupling agent KH-550 significantly improves the interfacial compatibility between composite particles and the aramid paper matrix, reducing internal defects within the composite aramid paper.
(2) When the content of K-BNNS composite particles is 10%, the composite aramid paper exhibits optimal comprehensive performance. Compared to Nomex paper, the breakdown voltage of partial discharge is increased by an average of 48.73%, thermal conductivity of plane thermal conductivity and through-plane is improved by 502.86% and 633.13%, respectively, and tensile strength is increased by 2.49%.
(3) The superior performance of F-10 composite aramid paper is attributed to two main reasons. First, a complete thermal conductive network is formed within the composite aramid paper matrix at this filler content, reducing matrix interfacial thermal resistance and enhancing the thermal conductivity of the material. Second, the addition of composite particles introduces numerous hydrogen bonds, strengthening the interfacial bonding force between the aramid matrix and composite particles. It indicates that the energy barriers for charge trapping and de-trapping are increased, charge migration at defect is inhibited, and the activity of partial discharge is suppressed.

## Figures and Tables

**Table 1:** The main materials.

Material Name	Specification	Manufacturer
Boron nitride (BN)	Particle size 1 μm	Aladdin reagent Co., Ltd. (Shanghai, China)
Polymeric m-phenylenediamine solution (PMIA)	Solid content 20%	Taihe New Material Group Co., Ltd. (Shandong, China)
N,N-dimethylacetamide (DMAc)	Analytical purity (AR)	China National Pharmaceutical Group Chemical Reagent Co., Ltd. (Shanghai, China)
Double-distilled water		Shuyang Kehong Trading Co., Ltd. (Jiangsu, China)
Polytetrafluoroethylene filter membrane	Aperture 0.1 μm	German Filter New Materials Technology Co., Ltd. (Zhejiang, China)
Silane coupling agent KH-550	Chemically pure	Nanjing Chuanshi Chemical Auxiliary Co., Ltd. (Nanjing, China)

**Table 2:** Thermal conductivity of different composite insulating papers.

Composite Aramid Paper	Composite Particle Content	The in-Plane Thermal Conductivity	The Through-Plane Thermal Conductivity
NBKP/h-BN [23]	40%	0.682W/(m·K)	
BNNS/ANF [24]	30%		5.31 W/(m·K)
(BNNS@PDA)/ANF [25]	50%	0.62 W/(m·K)	3.94 W/(m·K)
BTCN/PMIA [26]	10%	0.62 W/(m·K)	
D@BTW-fBNNSs [27]	15%	0.57 W/(m·K)	
Sample of this article	10%	0.76 W/(m·K)	7.64W/(m·K)

## Data Availability

The data presented in this study are available on request from the corresponding author.
